# An Easy-to-Use Arrayed Brain–Heart Chip

**DOI:** 10.3390/bios14110517

**Published:** 2024-10-22

**Authors:** Xiyao Peng, Lei Wu, Qiushi Li, Yuqing Ge, Tiegang Xu, Jianlong Zhao

**Affiliations:** 1 State Key Laboratory of Transducer Technology, Shanghai Institute of Microsystem and Information Technology, Chinese Academy of Sciences, Shanghai 200050, China; pengxiyao@mail.ustc.edu.cn (X.P.); liqiushi@mail.sim.ac.cn (Q.L.); yqge@mail.sim.ac.cn (Y.G.); xutiegang@mail.sim.ac.cn (T.X.); 2 College of Materials Science and Opto-Electronic Technology, University of Chinese Academy of Sciences, Beijing 100049, China

**Keywords:** brain–heart chip, fibrin, endothelial barrier, cerebral organoid, cardiac organoid

## Abstract

Multi-organ chips are effective at emulating human tissue and organ functions and at replicating the interactions among tissues and organs. An arrayed brain–heart chip was introduced whose configuration comprises open culture chambers and closed biomimetic vascular channels distributed in a horizontal pattern, separated from each other by an endothelial barrier based on fibrin matrix. A 300 μm-high and 13.2 mm-long endothelial barrier surrounded each organoid culture chamber, thereby satisfying the material transport requirements. Numerical simulations were used to analyze the construction process of fibrin barriers in order to optimize the structural design and experimental manipulation, which exhibited a high degree of correlation with experiment results. In each interconnective unit, a cerebral organoid, a cardiac organoid, and endothelial cells were co-cultured stably for a minimum of one week. The permeability of the endothelial barrier and recirculating perfusion enabled cross talk between cerebral organoids and cardiac organoids, as well as between organoids and endothelial cells. This was corroborated by the presence of cardiac troponin I (cTnI) in the cerebral organoid culture chamber and the observation of cerebral organoid and endothelial cells invading the fibrin matrix after one week of co-culture. The arrayed chip was simple to manipulate, clearly visible under a microscope, and compatible with automated pipetting devices, and therefore had significant potential for application.

## 1. Introduction

Multi-organ-on-a-chip is a biomimetic platform that utilizes microfluidic chip and three-dimension (3D) cell culture to model human physiology and pathology in vitro. Since the publication of a lung-on-a-chip in 2010 [1], this field has seen over a decade of development, resulting in various organ-specific chips, including liver-on-a-chip [2,3,4], intestine-on-a-chip [2,5,6,7], brain-on-a-chip [8,9,10], heart-on-a-chip [11,12], etc. These chips have already shown great potential for drug discovery and precision medicine.

In order to gain a deeper understanding of the interactions between tissues and organs, as well as their coordinated responses to external stimuli, it is essential to develop more sophisticated physiological and pathological models that incorporate interconnected organ communication networks [13,14]. One approach entailed the interconnection of multiple single-organ chips via small tubes, with pumps facilitating the flow of culture medium through the tubing to simulate blood flow, which enabled the exchange of substances and interactions among organs and tissues [15,16]. However, the introduction of external tubing led to an overall increase in the volume of fluid, which in turn resulted in a reduction in the concentration of organoid and tissue secretions within the microenvironment. This ultimately impeded the study of interactions between tissues and organoids. Another approach was to develop multi-organ chips, in which the channels and culture chambers were integrated into a single chip. Various tissues and organoids were cultured in designated culture chambers, which were interconnected through microfluidic channels [17].

To further emulate the niches of the interior and exterior of blood vessels, a vascular barrier structure was incorporated between the culture chamber and the perfusion channel. This structure permitted the permeation of compound molecules, ions, cytokines, and exosomes, thereby facilitating communications between chambers and channels [2,18,19,20]. The construction of vascular barriers frequently employed a “sandwich” structure, wherein the culture chamber was situated above the fluid channel, separated by a porous membrane or scaffold seeded with vascular endothelial cells. A notable limitation of this approach was its restricted capacity for microscopic imaging. During brightfield imaging, the dense pores of the membrane impeded the observation of cells growing on the membrane. Furthermore, the in situ observation of the chip was impeded by the fact that the membrane or scaffold was situated at a greater distance from the objective, which made it challenging to distinguish between the different cells that were growing on either side of the membrane and to conduct high-resolution imaging of the tissues that were located above the membrane or scaffold. Consequently, the real-time observation and detection of processes such as the traversal of nanoparticles, immune cells, or circulating tumor cells across the barrier proved to be an arduous task.

To address these imaging challenges, the culture chambers and biomimetic blood vessels can be arranged in a horizontal pattern, with a hydrogel “wall” functionalized with endothelial cells serving as the vascular barrier. MIMETAS has developed chips featuring several parallel, enclosed channels, one of which is filled with type I rat tail collagen [21,22]. This hydrogel-filled channel communicates with adjacent parallel channels but is confined by raised “phaseguide” structures that prevent the hydrogel from overflowing into the side channels. Endothelial cells are then introduced to form an endothelial layer supported by the hydrogel wall. Other researchers have also utilized hydrogel in the construction of vascular network. A fibrin matrix was constructed in a gap in the bottom of the culture chamber and was connected to the culture medium reservoirs [23]. The open culture chambers could accommodate organoids, while human umbilical vein endothelial cells (HUVECs) and human lung fibroblasts within the fibrin could form capillary networks under the influence of cytokines and shear stress, thereby simulating the capillary microenvironment of the organoids.

The brain and the heart are two of the most vital organs in the human body. The study of communication between brain and heart is significant for the advancement of our understanding of disease. Recently, researchers have identified the persistence of pro-inflammatory changes in monocytes/macrophages in multiple organs, particularly the heart, following a stroke. The changes have been linked to the development of cardiac fibrosis and dysfunction. This phenomenon has been observed in both mice and stroke patients. It has been demonstrated that IL-1β plays a pivotal role in the epigenetic alterations associated with innate immune memory [24]. Additionally, damage to the endothelium of blood vessels has the potential to impact the brain and heart. Endothelial cells exhibit high secretory and pro-inflammatory properties in response to external injurious stimuli, and the pro-inflammatory cytokines and chemokines they produce transmit injury signals to parenchymal organs [25].

This paper introduced an arrayed brain–heart chip comprising a biomimetic vascular channel that encircles the open organoid culture chambers. In each unit, a cerebral organoid and a cardiac organoid were, respectively, cultured in two separate open culture chambers. A fibrin matrix wall was positioned around the perimeter of the culture chamber, with HUVECs cultured on the external surface of the wall. The outer channels were designed to emulate the blood vessels, and perfusion flow was maintained through a rocking shaker. The perfusion method has been utilized for a high-throughput blood–brain barrier chip [26]. The arrayed chip design permitted both automated pipetting and microscopic imaging. The numerical calculations were used to direct experimental optimizations, including surface hydrophilic treatment and the regulation of the loading volume of fibrinogen. Furthermore, they were utilized to guide the structural design of the convergence angle between the biomimetic vascular channel and the hydrogel channel. The morphology and permeability of the endothelial barrier were subsequently investigated. The utilization of this chip facilitated the co-culture of cerebral and cardiac organoids with an endothelial barrier and demonstrated the feasibility of investigating inter-organoid communication.

## 2. Materials and Methods

### 2.1. Fabrication and Assembly of the Chip

The polydimethylsiloxane (PDMS) structure was fabricated using soft lithography as described previously [27], and briefly described as follows. First, SUEX^®^ Thick Dry Film Sheets (DJ Microlaminates) with thicknesses of 300 μm and 500 μm were sequentially transferred onto a silicon wafer. Photolithography was then performed using a maskless lithography machine (MicroWriter ML3, Durham Magneto Optics, Cambridge, UK) at energy densities of 12,000 J/cm^2^ and 20,000 J/cm^2^, respectively, to create a two-level master on a silicon wafer. Subsequently, a PDMS mixture (base elastomer: curing agent = 10:1 *w*/*w*, Sylgard 184, Dow Corning, Midland, MI, USA) was poured onto the SU8 mold and cured at 60 °C for 4 h. The culture chambers and vascular channel inlets and outlets were punched out on the cured PDMS. The PDMS was then irreversibly bonded to plasma-treated glass slides and then sterilized at 120 °C using an autoclave. At last, the ultraviolet-sterilized polymethylmethacrylate (PMMA) was securely attached to the bonded chip, aligning the through-holes in the PMMA with the culture chambers and inlets/outlets of the vascular channel, thus increasing the liquid storage capacity.

### 2.2. Simulation of Fibrinogen Advancing in the Chip

The numerical simulations in this study were conducted using COMSOL Multiphysics 6.2 software. The spontaneous advancement of the aqueous phase within the chip microchannels was modeled as a two-phase flow problem. A 3D model was constructed for this purpose, utilizing the “Laminar Two-Phase Flow, Phase Field Interface” physics in the “Fluid Flow” module. At 0 °C, the viscosity and surface contact angle of the fibrinogen solution with the chip are approximately equal to that of water. Therefore, the aqueous phase in the simulation was defined as water. The Navier–Stokes equations and phase field equations were solved to track the interface between the water and air phases. The computational domain was divided into two regions: a rectangular region filled initially with water, while the rest was filled with air. The boundary conditions for the simulation were set as follows: (i) the initial velocities of both the water and air phases were set to 0 mm/s; (ii) the initial pressure of the water, both at the inlet and outlet, was set to the standard atmosphere; (iii) all chip walls were assigned no-slip boundary conditions and assumed to have identical contact angles and hydrophilicity. The simulations were based on the geometric parameters of the PDMS chip. In the phase field settings, the interface thickness parameter was set to 6.5 × 10⁻^5^ m, and the mobility tuning parameter was set to 50 m·s/kg. Additionally, the following assumptions were made while constructing the two-phase flow model: (i) The water phase was modeled as a homogeneous, incompressible Newtonian fluid with a density of 998 kg/m^3^ and a viscosity of 1.0 × 10⁻^3^ Pa·s. The air phase had a density of 1.2 kg/m^3^ and a viscosity of 1.8 × 10⁻^5^ Pa·s. (ii) No energy exchange or chemical reactions occurred between the computational domain and its surroundings. (iii) Gravitational effects were neglected.

### 2.3. Simulation of Perfusion Flow Field

To evaluate the perfusion within the chip, flow field simulations were also carried out using COMSOL Multiphysics. The flow field modeling was based on the incompressible Navier−Stokes equations and employed the “Laminar Flow” interface. In this simulation, the computational domain was treated as three-dimensional. The boundary conditions were set as follows: a pressure boundary condition was applied at the inlet, the outlet was maintained at zero pressure, and no-slip boundary conditions were applied to all walls.

### 2.4. Method for Construction of the Fibrin Matrix Barrier

A working solution of fibrinogen (20430ES, Yeasen Biotechnology, Shanghai, China) with a concentration of 10 mg/mL and thrombin (T8021, Solarbio, Beijing, China) with an activity of 20 U/mL was prepared in advance. The assembled chip was subjected to plasma treatment (200 W, 2 min) and then placed on ice for 2 min. Subsequently, thrombin and fibrinogen solutions were mixed at a 1:4 ratio, and 10 μL of the mixed solution was dropped onto each hydrogel inlet. The chip was then placed in a 37 °C incubator for 30 min to allow the hydrogel to fully polymerize. After polymerization, phosphate-buffered saline (PBS, pH7.4, Thermo Fisher Scientific, Waltham, MA, USA) was added to the chip, which was placed in 37 °C incubator for 12 h to allow for complete swelling and then stored in a clean, moist environment until needed.

### 2.5. Perfusion Culture of HUVECs and HUVEC-RFPs

HUVECs and HUVECs transfected with red fluorescent protein (HUVEC-RFPs) were obtained from Wuxi Puhe Biomedical Technology Co., Ltd. (Wuxi, China) and were used between passages 4 and 8. All cell types were cultured in EGM-2 (C3162, LONZA, Basel, Switzerland) in an incubator at 37 °C with 5% CO_2_. Cells were trypsinized, centrifuged, and resuspended to a concentration of 1 × 10^7^ cells/mL.

The chip, with the fibrin barrier already constructed, was prepared by removing the PBS and injecting 50 μL of the cell suspension into the channels of the chip. The chip was then placed in a 37 °C incubator with 5% CO_2_ for culture. After 12 h, 200 μL EGM-2 was added in the inlet. The chip was then placed on a rocking shaker (IBAC ROCKER, Daxiang Bio, Beijing, China) for perfusion culture. The rocker was set with the following parameters: tilt angle of 10°, swing speed of 20 cycles/min, and a delay of 2 s between swings. Media was replaced daily, and cell growth and behavior were observed and recorded.

### 2.6. Measurement of Barrier Permeability

To measure the permeability of the barrier, a syringe pump was used to perfuse FITC -dextran solutions with concentrations of 1 mg/mL and molecular weights of 4 kDa and 70 kDa (46944, 46945, Merck, Darmstadt, Germany), respectively, into the chip’s perfusion vascular channel at a flow rate of 20 μL/min. To ensure that the barrier was saturated with the fluorescent solution, no solution was added to the culture chambers initially. After 15 min of perfusion, one of the culture chambers of the brain–heart chip was washed 1–2 times with PBS. To facilitate the collection of fluorescent molecules permeating the barrier, 50 μL of PBS was added to the culture chamber. And then the perfusion of the fluorescent solution was resumed. One minute later, 50 μL of the solution from the culture chamber was transferred to a black 96-well plate. An equal volume of PBS was added back to the culture chamber for the next sampling. Fluorescence intensity was measured using a microplate reader (Spark, Tecan, Mannedorf, Switzerland) and compared with a standard curve. Barrier permeability (*P*) was calculated using the equation:

(1) $$P=\frac{\left(C_{1}-C_{0}\right)\times V}{C\times A\times t}$$

where *C*_1_ and *C*_0_ is the concentration of the sample and the initial solution, respectively, in the culture chamber, *C* is the concentration of perfusion solution, *V* is the volume of solution in the culture chamber, *A* is the area of the barrier, and *t* is the perfusion time.

### 2.7. Organoid Induction and Characterization

Human-induced pluripotent stem cells (iPSCs) (DYR0100, National Collection of Authenticated Cell Cultures, Shanghai, China) were used to induce cerebral and cardiac differentiation.

The induction of cerebral organoid was performed using the STEMdiff™ Cerebral Organoid Kit (08570, 08571, STEMCELL, Vancouver, BC, Canada) according to the provided protocol. Briefly, iPSCs, at a density of 9000 cells per well, were first cultured to form embryoid bodies (EBs) in a 96-well low-attachment U-bottom plate, starting on Day 0 (D0). On Days 2 and 4, 100 μL of EB formation medium was added to each well. Induction began on Day 5 (D5). When dense centers and radial translucent bands were observed in the EBs on Day 7 (D7), the EBs were embedded in Matrigel (354277, Corning, NY, USA) and cultured on a shaker with expansion medium. On Day 10 (D10), the expansion effect was assessed, and if extensive neuroepithelium was observed as evidenced by budding of the organoid surface, the medium was replaced with maturation medium. Media were exchanged every two days, and the culture was continued on a shaker for at least 10 additional days.

Cardiac organoids were derived using the Human iPSC-Derived Cardiac Organoid Differentiation Kit (RIPO-HWM002K, ACRObiosystems, Newark, DE, USA) following the provided protocol. Briefly, iPSCs, at a density of 7500 cells per well, were cultured to form EBs in a 96-well low-attachment U-bottom plate. The formation of EBs was assessed the following day, and EBs with clear, smooth edges and sizes between 300 and 500 μm were deemed suitable for further differentiation. Differentiation started on Day 0 (D0). After removing all media, 200 μL of medium A was added to each well and the culture was incubated for 48 h. Following this, the medium was replaced with medium B, which was changed every 24 h for a total of 4 changes. Subsequently, medium C was introduced, with changes every 24 h for a total of 5 changes. After this period, all cardiac organoids were transferred to a low-attachment 6-well plate and cultured with medium M-M on a shaker. On Day 10 (D10), pulsating cardiac organoids, indicating maturity, were observed.

Organoids were characterized using immunofluorescence to label specific proteins to confirm organoid identity (Figure S1). To examine the transport of cardiac troponin I between two organoid culture environments, experiments were conducted using cardiac organoids with impaired cardiomyocytes, which exhibited reduced or ceased pulsation.

### 2.8. Organoids and Endothelial Cells Co-Culture in the Chip

A suspension of endothelial cells at a concentration of 1 × 10^7^ cells/mL (50 μL) was introduced into the brain–heart chip. After three days of perfusion culture according to 2.5, the endothelial barrier was fully formed. Then, cerebral and cardiac organoids were placed in the two culture chambers, respectively, for further co-culture.

The cardiac medium M-M, cerebral maturation medium, and EGM-2 medium were introduced to the cardiac chamber, cerebral chamber, and perfusion channel inlet, with a volume of 200 μL each. The chip was then placed on a rocking shaker, which was set with the following parameters: tilt angle of 10°, swing speed of 20 cycles/min, and a delay of 2 s between swings. The culture media in each compartment were completely replaced every 24 h.

### 2.9. Cardiac Troponin I (cTnI) Measurement

After 3 days of co-culture, the media were replaced, and the culture was continued for an additional 12 h. Subsequently, 100 μL of solution was collected from the cardiac and cerebral chambers, respectively. cTnI levels were measured using a fluorescent immunoassay analyzer (Getein1100, Getein Biotech, Nanjing, China). The fresh complete medium corresponding to the organoid was used as a blank control. The final value was obtained by subtracting the blank control value from the measured values.

### 2.10. Characterization of Fibrin Barrier and Endothelial Barrier

Laser confocal microscopy 3D imaging was used to observe fibrin barriers constructed in chips. First, 50 μL of FITC (46950, Merck) fluorescent solution, at concentration of 100 μg/mL, was added to both the perfusion channel and the culture chamber and incubated for 10 min to facilitate the dyeing of the fibrin matrix. The fluorescent solution was then removed, and the chip was washed twice with an equal volume of PBS. The chip was then observed under a confocal microscope (TCS SP8, Leica, Wetzlar, Germany) with z-stack scanning performed to reconstruct a 3D image.

The morphology of the endothelial barrier on the surface of the fibrin matrix was characterized by cytoskeleton staining. Endothelial cells were fixed with 4% paraformaldehyde solution for 30 min, followed by two washes with PBS. TRITC-Phalloidin (EFL-FA-001, EFL-tech, Suzhou, China) diluted 1:1000, was added to the chip for 30 min to stain the F-actin. The chip was then washed twice with PBS. The stained chip was similarly imaged using a confocal microscope, with z-stack scanning and 3D reconstruction for further analysis.

## 3. Results and Discussion

### 3.1. Chip Design and Fabrication

**Figure 1:**
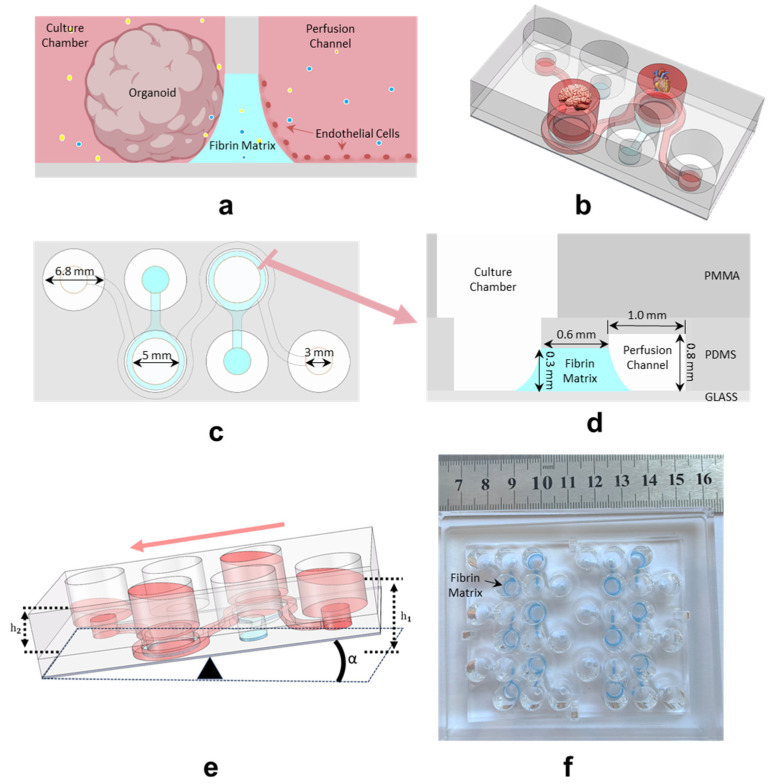
Diagram of the brain–heart chip. (**a**) In each culture unit, a cerebral organoid/a cardiac organoid and endothelial cells were cultured on each side of the hydrogel, with cross talk occurring across the barrier. (**b**) A perfusion channel as a biomimetic blood vessel connects two independent culture chambers in which a cerebral organoid and a cardiac organoid are grown. (**c**) Top view of the chip. (**d**) Cross-section of chip at marking. Not exactly to scale. (**e**) Gravity perfusion using a rocking shaker. (**f**). Photograph of the arrayed brain–heart chip with colored fibrin matrix barriers.

In the human body, different organs and tissues exist within relatively independent microenvironmental niches, and inter-organ communications primarily occurs through the circulatory system. The establishment of a barrier interface between organoids and biomimetic blood vessels is a crucial step in the development of highly biomimetic multi-organ chips. In the conventional approach to multi-organ chips, a porous membrane at the base of organoid culture chambers was used to construct endothelial barriers. In contrast, this study proposed a novel barrier structure, where organoids and endothelial cells were positioned on opposite sides of a hydrogel matrix, with the endothelial cells forming a monolayer on the hydrogel walls (Figure 1a). On the endothelial side, a biomimetic blood vessel facilitated perfusion, connecting different organoid culture chambers (Figure 1b). This biomimetic barrier maintained distinct microenvironments for different organoids while allowing molecular permeation across the barrier, thereby enabling interaction between the organoids and endothelial cells, as well as between different organoids. The horizontal distribution design of the flow channels and culture chambers also allowed for easy microscopic observation.

To improve uniformity in the culture chamber, increase the area of the vascular barrier, and facilitate automated operations, the biomimetic vascular channel was designed in a circular shape, as shown in Figure 1c. A clear cross-sectional view of the chip is revealed in Figure 1d, displaying its three-layer structure. The substrate was made of highly transparent glass, while the PDMS structure was bonded to the glass substrate, forming the culture chambers and channels. The fibrin matrix barrier was constructed within the gap between the PDMS and the glass. The PDMS inlets and culture chambers were aligned with the through-holes in the PMMA layer. These through-holes followed the standard layout of a 96-well plate and were designed to expand the culture chambers and inlets and outlets of the channels to hold more media for long-term culture and to allow perfusion with a rocking shaker. The height and width of the fibrin matrix barrier are 300 μm and 600 μm, respectively. This configuration effectively isolated the culture chambers from the vascular channel while maintaining molecular permeability. The physical properties of natural hydrogels, such as mechanical properties, high water absorption, and chemical composition, mimic the extracellular matrix and are therefore able to support the adhesion of cells or organoids on the barrier [28].

The bidirection flowing of media in biomimetic vascular channels by rocking shaker is shown in Figure 1e. The swing angle α of the shaker determined the height difference between the inlet and outlet liquid levels, resulting in a pressure difference between the inlet and outlet that caused media to flow. The chip was arranged in a 2 × 3 configuration, with an overall size equivalent to half of a standard 96-well plate, as shown in Figure 1f. This allowed the chip to be compatible with 96-well plate shakers (Figure S2a). In addition, due to its open design, the chip array was compatible with most commercial automated pipetting systems (Figure S2b).

### 3.2. Construction of the Fibrin Matrix Barrier

**Figure 2:**
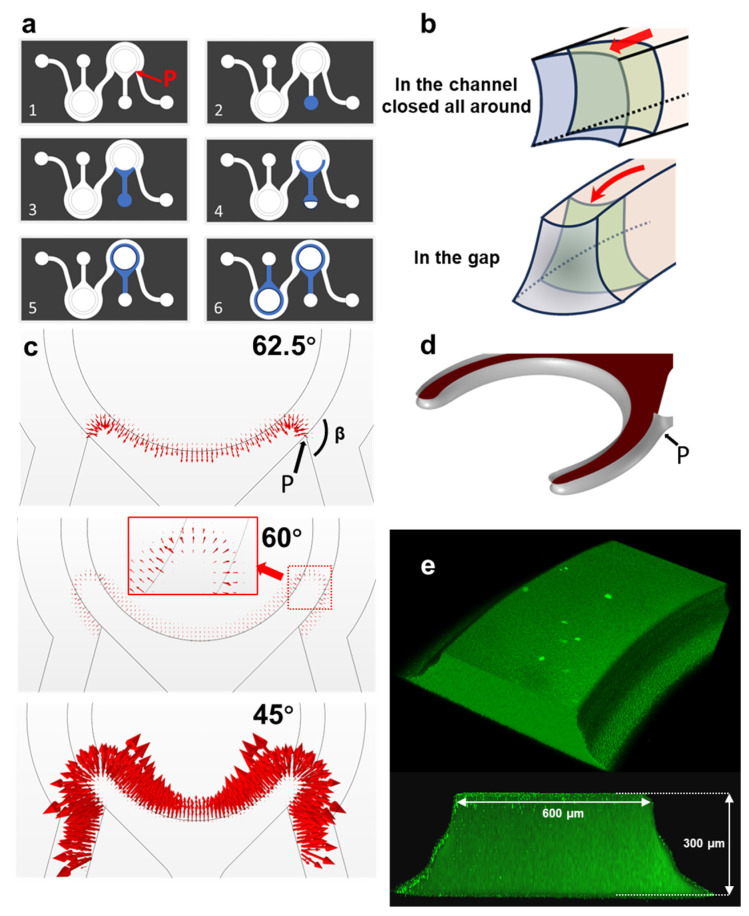
(**a**) Diagram of the hydrogel perfusion process. P is the point of convergence between the perfusion vascular channel and the hydrogel channel. (**b**) Illustrations of the hydrogel advancing in the channels closed all around and in the gap of the hydrogel channel, respectively. (**c**) The force distributions of the gas–liquid interface when liquid advancing under different surface contact angle conditions with COMSOL simulation. β is the convergence angle between the perfusion vascular channel and the hydrogel channel. (**d**) Simulation of hydrogel advancing in the gap. (**e**) Three-dimensional and cross-sectional views of the fibrin barrier using confocal microscopy.

When the unpolymerized fibrinogen flowed through the chip, it was subjected to both capillary and viscous forces. The fibrinogen solution at 0 °C exhibited a flow behavior analogous to that of water, suggesting that capillary forces exert a dominant influence on the flow. The process of hydrogel barrier formation is shown in Figure 2a. First, the hydrogel was added to the inlet of a hydrogel channel (Figure 2a(1,2)) and the liquid was drawn into the chip channels by capillary action. It then split into two streams, reaching the junction point P and moving through the gap between the channel and the culture chamber (Figure 2a(3)). The streams continued along the gap (Figure 2a(4)) and eventually merged to form a complete circle (Figure 2a(5)). This method allowed the construction of a fibrin barrier around the culture chamber, creating two relatively independent regions for organoid culture (Figure 2a(6)). The flow of the liquid was primarily determined by the channel geometry and the contact angle between the hydrogel and the solid surface, which is analyzed in detail below.

According to the Cassie-Baxter equation [29]:

(2) $${\cos\theta }^{*}=\sum_{i}f_{i}{\cos\theta }_{i}$$

where $\theta^{*}$ is the effective contact angle of the entire system, and *f_i_* is the normalized coefficient of contact surface, with $\sum_{i}f_{i}=1$. If *f_SL_* and *f_GL_* represent the normalized coefficients of solid–liquid and gas–liquid contact areas, respectively, then Equation (2) becomes

(3) $${\cos\theta }^{*}=\sum_{i}f_{SLi}{\cos\theta }_{i}+f_{GL}\cos\pi$$

If the liquid has the same contact angle θ on the solid surface, Equation (3) simplifies to

(4) $${\cos\theta }^{*}=f\cos\theta -\left(1-f\right)$$

For the liquid to advance spontaneously, it must satisfy

(5) $${\cos\theta }^{*}>0$$

In the chip shown in Figure 2a, the fluid flowing in the hydrogel channel was divided into two processes, as shown in Figure 2b. Before reaching the point P (Figure 2b, top), the liquid flowed in a rectangular, closed all around channel where the contact angle $\theta^{*}=\theta$, and as long as the surface contact angle was less than 90°, surface tension would drive the liquid to the point P. After the liquid passed through P (Figure 2b, bottom), the channel opened on both sides, with the upper side having a width of 0.6 mm and the lower side being an unbounded plane. At this point, the solid–liquid surface tension driving the liquid was significantly reduced, while the gas–liquid surface tension resisting the flow increased sharply. According to Equations (4) and (5), a smaller contact angle was required for the liquid to continue advancing along the top wall. Furthermore, due to the unbounded nature of the bottom wall, the liquid could also overflow from point P into the adjacent vascular channel.

To derive reference values of $\theta^{*}$, the process of hydrogel advancement was simulated using COMSOL Multiphysics 6.2 software (Figure 2c,d). Figure 2c shows the forces acting on the gas–liquid interface at different surface contact angles. The size and direction of the arrows represent the magnitude and direction of the forces. When the contact angle is 62.5°, the resultant force at the interface is negative, i.e., the direction of the resultant force is pointing from the air to the liquid. When the contact angle is reduced to 60°, the resultant force becomes positive. At 45°, the positive driving force at the interface is significantly greater, and there is also a driving force along the vascular channel wall, which explains the overflow at the point P, as shown in Figure 2d. When the liquid filled the gap along the top wall, the liquid overflow from the point P forms only a thin layer on the wall, which has a minimal impact on the experiment.

The contact angles of fibrinogen on plasma-treated glass and PDMS were nearly identical, measuring 16.5° and 17.3° at 0 °C, respectively (Figure S3). The flow behavior in this case was similar to the simulated results for a 45° surface contact angle. Under these conditions, fibrinogen solution could spontaneously fill the gap around the culture chamber driven by capillary forces, forming a nearly trapezoidal advancing front. At the same time, some liquid could overflow at point P, making it necessary to control the total volume of hydrogel loaded to prevent further filling of the vascular channel. Finally, a mixture of 10 μL of fibrinogen and thrombin was used to create the fibrin matrix barrier, as shown in the confocal image in Figure 2e. The meniscus of the gel barrier on the culture chamber side and the channel side was not completely symmetrical due to the difference in inner and outer radii. Given that the same amount of surface tension work was performed on both sides, the inner circle advances further than the outer circle. The Video S1 of the experiment in which the fibrinogen solution spontaneously filled the hydrogel channel driven by capillary forces can be found in Supplementary Information.

### 3.3. Influence of the Convergence Angle of Vascular Channel on Hydrogel Overflow

**Figure 3:**
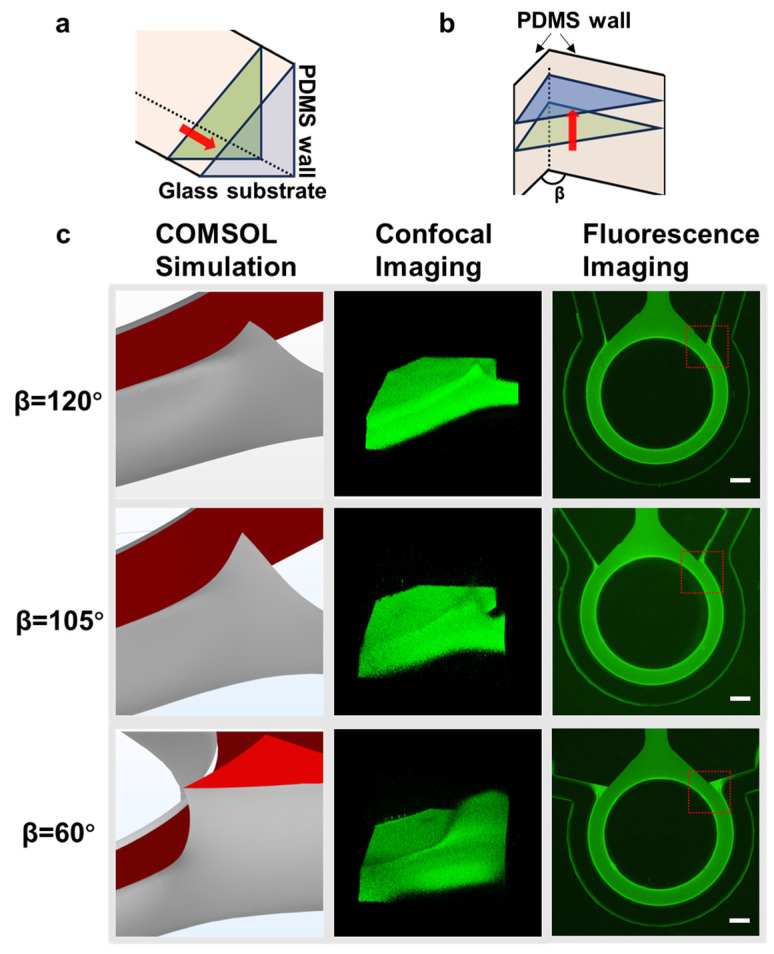
Hydrogel overflow analysis after passing through the P point. (**a**,**b**) Simplified schematic of the overflow hydrogel flowing along the right-angle corner formed by the glass substrate and the PDMS wall, and along the angle β corner formed by the two PDMS walls in the perfusion channel. (**c**) Under different β conditions, COMSOL simulation, laser confocal imaging, and fluorescence imaging were used to analyze the overflow of hydrogel near the P-convergence point. The plots in the leftmost two columns represented enlargements of the area delineated in red in the rightmost column of plots. Scale bars, 1 mm.

According to the previous simulation results, if the hydrogel solution could pass through the boundary point P and continue to flow forward, it would be difficult to avoid overflow into the adjacent vascular channel. Once overflow occurred, the fluid could continue to flow either horizontally (Figure 3a) or upward along the channel walls (Figure 3b). With a fixed surface contact angle, the flow behavior was influenced by the structure of the flow channels. The effect of the convergence angle between the vascular channel and the hydrogel channel on overflow was investigated through simulations and experiments as follows.

The convergence angle, β, formed by the vascular channel and the hydrogel channel, as marked in Figure 2c, was the primary variable affecting the overflow. To study this phenomenon, we set a surface contact angle of 45° and tested three different convergence angles: 120°, 105°, and 60°, under the same conditions.

As shown in Figure 3c, the first column depicted simulations near the boundary point P, the second column presented confocal imaging in experiments, and the third column showed the complete barrier morphology observed under a microscope. When the convergence angle was 120°, the hydrogel showed a slight upward tendency at the boundary point P, which is consistent with the confocal imaging. The full view of the hydrogel barrier in the chip indicated minimal accumulation of hydrogel near point P. With a convergence angle of 105°, the upward movement of the liquid became more pronounced, again consistent with the confocal imaging. There was increased hydrogel accumulation near point P. When the angle was reduced to 60°, significant changes were observed. The hydrogel moved upward along both PDMS walls of the channel, reaching the top of the channel and forming a triangular prism-like accumulation, which was consistent with the confocal imaging and the complete barrier morphology.

The above analysis showed that overflow was inevitable, but by properly designing the convergence angle and controlling the amount of hydrogel, the effect of overflow could be minimized while ensuring the formation of a complete hydrogel barrier. In the final chip design, the convergence angle was fixed at 120°.

### 3.4. Endothelial Cell Perfusion Culture and Morphological and Functional Characterization of the Endothelial Barrier

**Figure 4:**
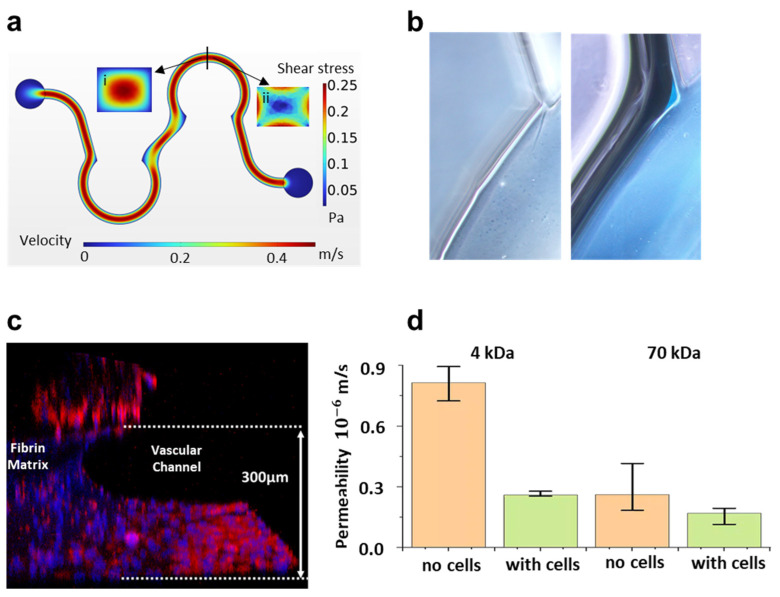
Characterization of the biomimetic vascular channel. (**a**) Flow field distribution in the channel. The insets in the figure show the flow field distribution (i) and shear stress distribution (ii) of the cross section at the marking, respectively. (**b**) The detailed micrographs near point P without (**left**) and with (**right**) the colored hydrogel barrier. (**c**) Nuclei and F-actin of endothelial cells cultured on the fibrin matrix were fluorescently stained and characterized by confocal microscopy. (**d**) FITC-Dextran permeability of the barriers without and with the HUVECs. Data are shown as mean ± SD and *n* = 3.

In the human body, communication between tissues and organs is facilitated by the circulatory system. In an organ-on-a-chip, media flow in the biomimetic blood vessel plays a critical role in maintaining the physiological function of endothelial cells and facilitating interactions between tissues and organs. A rocking shaker was used to generate perfusion in the biomimetic vascular channel of the arrayed brain–heart chip (Figure S2a). The distance between the inlet and outlet reservoirs along the rocking axis of the shaker was 27 mm, and the maximum tilt angle was 10°. Under these conditions, the maximum pressure difference (ΔP) between the inlet and outlet reservoirs is 45.9 Pa. We used COMSOL to study the flow field distribution according to the pressure difference. The flow in the biomimetic vascular channel with the hydrogel barrier was a laminar flow with a uniform flow velocity, as shown in Figure 4a. The hydrogel barrier was subjected to a maximum shear stress of 0.253 Pa.

In the simulation, the computational domain of the biomimetic vascular channel adopted the design structure of the chip. However, the hydrogel slightly changed the structure of the channel. Figure 4b showed that the hydrogel overflow helped to smooth the sharp corner, thus reducing the dead volume at this location in the chip. This indicated the beneficial role of the hydrogel in minimizing irregular flow within the channels.

HUVECs were introduced into the chip and perfusion cultured on the surface of the fibrin matrix. After 2 days, the endothelial barrier morphology was observed by cytoskeleton fluorescence staining. As shown in Figure 4c, endothelial cells were observed to cover the bottom of the vascular channel and the fibrin barrier. Some cells were observed to ascend to a height exceeding that of the barrier, reaching the PDMS walls. Due to the barrier’s height of 300 μm and the limited penetration depth of the confocal microscope, the fluorescence intensity of the stained cells on the curved fibrin surface was relatively weak.

To allow the long-term observation of endothelial cell growth on the hydrogel barrier, HUVEC-RFPs were used in later experiments. In the absence of organoids, endothelial cells were able to grow on the fibrinogen barrier for more than two weeks without invading the fibrin matrix.

Two different molecular weights of dextran-FITC were used to analyze the permeability of the barrier after functionalization by endothelial cells. Figure 4d shows that the fibrin barrier exhibited good permeability to molecules. Notably, the permeability of the barrier with endothelial cells for 4 kDa dextran was less than one-third that of the barrier without cells, suggesting that the endothelial monolayer played a more dominant role in the crossing of the barrier by small molecules compared with the hydrogel layer. The permeability of the vascular barrier in this study was comparable to that constructed using porous membrane scaffolds [20].

### 3.5. Co-Culture and Communication of Organoids

In the brain–heart chip, cerebral and cardiac organoids were cultured in relatively independent culture chambers, which allowed the application of organoid-specific media and the maintenance of distinct microenvironments. The excellent biocompatibility and molecular permeability of the biological hydrogel favored cell growth and allowed cytokines transportation between organoids.

**Figure 5:**
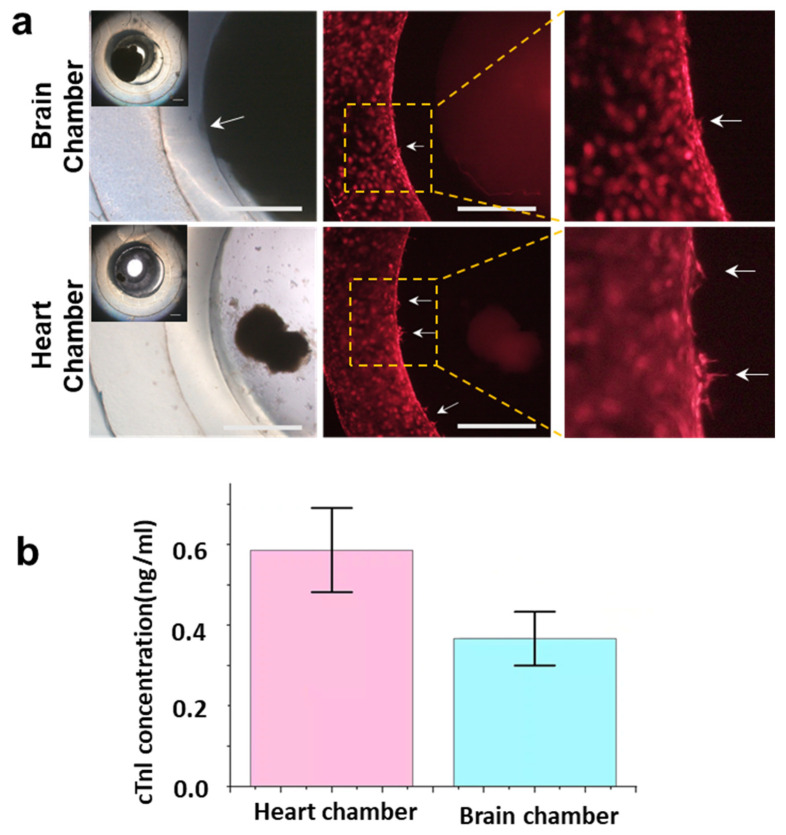
Co-culture and communication of different organoids and endothelial barrier in the brain–heart chip. (**a**) Bright field and fluorescence micrographs. The insets are a full view of each culture chamber and its surrounding vascular channel. Arrows indicate the location of the cerebral organoid and the endothelial cells invasion. Scale bars, 1 mm. (**b**) Concentration of cTnI within the two chambers 12 h after medium exchange. Data are shown as mean ± SD and *n* = 3.

To observe the communications between the organoids, cardiac organoids that had lost their normal beating function after three weeks of culture were used. The cerebral organoids, the cardiac organoids, and HUVEC-RFPs can be cultured long-term within the chip. In Figure 5a, after seven days of co-culture, a small number of dissociated cells were observed in the cardiac organoid chamber, while the cerebral organoid remained intact and adhered to the fibrin barrier. The swinging of the rocking shaker caused the organoids to move, while the fibrin barrier provided an attachment wall for the organoids. The large-sized cerebral organoid adhered easily to fibrin because it moved slowly and had a large contact area with fibrin. In the biomimetic vascular channels, HUVEC-RFPs not only completely covered the fibrin barrier, forming bright edges in the picture, but a few cells also began to invade the fibrin (as indicated by arrows in Figure 5a), especially near the cardiac organoid chamber. A small portion of the cerebral organoid also started to infiltrate the fibrin. However, when HUVEC-RFPs were cultured alone in the chip for two weeks, no cell invasion into the fibrin was observed (Figure S4), suggesting an interaction between the endothelial cells and organoids.

Due to myocytes damage, the characteristic functional protein, cTnI, was released into the culture environment. After a media change, cTnI was detected in both culture chambers after 12 h (Figure 5b), with a slightly higher concentration in the cardiac organoid chamber (0.59 ± 0.10 ng/mL) compared with the cerebral organoid chamber (0.37 ± 0.07 ng/mL), indicating that biomolecules secreted by the cardiac organoid were able to cross the fibrin barrier twice and act on the cerebral organoid, demonstrating the chip’s potential for studying communications between organoids.

## 4. Conclusions

The arrayed brain–heart chip developed in this study, with its biomimetic vasculature surrounding the organoid culture chambers, partially recapitulated the in vivo interconnected multi-organ environment. The structure is horizontally distributed, facilitating microscopic observation. The endothelialized hydrogel barrier not only maintained the specific culture media required by different organoids but also facilitated communication between the organoids. The cerebral and cardiac organoids could be stably co-cultured in the chip for at least one week.

The chip design was simple, and the construction of the hydrogel barrier was straightforward. Once the hydrogel was added to the sample inlet, capillary action completed the construction of the barrier with a success rate approaching 100%, making it highly scalable. The chip array was compatible with the standard 96-well plate layout, facilitating integration with automated liquid handling systems. In addition, the rocking shaker perfusion method allowed for high-throughput processing, making the chip promising for high-throughput drug screening.

During the experiment, it was observed that after one week of co-culture of organoids and endothelial cells, both endothelial cells and cerebral organoids began to penetrate the fibrin. Therefore, if a longer-term in vitro brain–heart connection model is required, alternative hydrogel materials may need to be explored. Furthermore, previous studies have demonstrated angiogenesis within the fibrin hydrogel [21], suggesting that future studies could incorporate angiogenesis within the current hydrogel barrier to construct bioinspired vascular channels connecting the culture chambers and perfusion channels.

This chip had a modular design that could be easily expanded to create more interconnected organ chips as needed, providing versatile in vitro models for physiological and pathological studies. In the future, the chip could also integrate microelectrode arrays to monitor the electrical activity of electroactive organoids, further expanding its application potential.

## Data Availability

The data presented in this study are available upon request from the corresponding author.

## Supplementary Materials

The following supporting information can be downloaded at: https://www.mdpi.com/article/10.3390/bios14110517/s1, Figure S1: Immunofluorescence characterization of organoids. (a) Staining for brain regions and neuronal cell identities including neurons (DCX), neural stem cell progenitors (SOX2) and pre-plate/deep-layer neurons (TBR1). (b) Staining for cardiomyocytes (CTNT). Samples in a and b are respectively 45 days for and 15 days after initiation of the protocols. Scale bars, 300 µm (a) and 100 µm (b); Figure S2: Schematic diagram of the brain-heart chip array peripherals. (a) A rocking shaker for perfusion. (b) An automated pipetting platform for chip sampling; Figure S3: Contact angle measurement of a fibrinogen solution on the surfaces of plasma-treated glass and plasma-treated glass PDMS, respectively; Figure S4: Fluorescence micrograph of HUVEC-RFP grown in the chip for two weeks. Scale bar, 1mm. Video S1: Video of fibrinogen solution spontaneously filled the hydrogel channel.
