# Supplementary material for: An Easy-to-Use Arrayed Brain–Heart Chip
Source: Biosensors (Basel). 2024 Oct 22;14(11):517. doi: 10.3390/bios14110517 (PMC11592345; doi:10.3390/bios14110517)
Supplement: Supplementary file 1 [file biosensors-14-00517-s001.zip › biosensors-3237544 - SM to XML.pdf]

biosensors

Supplementary Material

# An Easy-to-Use Arrayed Brain–Heart Chip

**Xiyao Peng [1,2], Lei Wu [1,2,*], Qiushi Li [1], Yuqing Ge [1,2], Tiegang Xu [1] and Jianlong Zhao [1,2,*]**

[1] State Key Laboratory of Transducer Technology, Shanghai Institute of Microsystem and Information Technology, Chinese Academy of Sciences, Shanghai 200050, China; pengxiyao@mail.ustc.edu.cn (X.P.); liqiushi@mail.sim.ac.cn (Q.L.); yqge@mail.sim.ac.cn (Y.G.); xutiegang@mail.sim.ac.cn (T.X.)
[2] College of Materials Science and Opto-Electronic Technology, University of Chinese Academy of Sciences, Beijing 100049, China
* Correspondence: wulei@mail.sim.ac.cn (L.W.); jlzhao@mail.sim.ac.cn (J.Z.); Tel.: +86-62511070 (L.W. & J.Z.)

## 1. Immunofluorescence characterization of organoids

The cerebral and cardiac organoids were characterized by immunofluorescence. Neurons, neural stem cell progenitors and pre-plate/deep layer neurons were observed in different layers of the cerebral organoids (Figure S1a). Cardiomyocytes were observed in the surface layer of the cardiac organoids (Figure S1b).

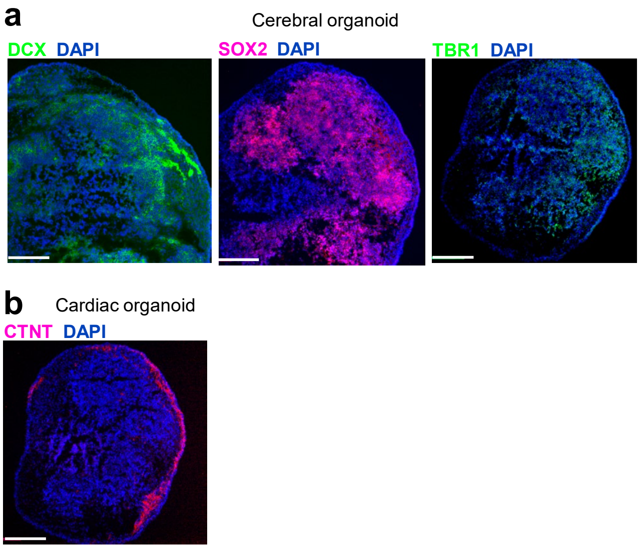


**Figure S1.** Immunofluorescence characterization of organoids. (a) Staining for brain regions and neuronal cell identities including neurons (DCX), neural stem cell progenitors (SOX2) and pre-plate/deep-layer neurons (TBR1). (b) Staining for cardiomyocytes (CTNT). Samples in a and b are respectively 45 days for and 15 days after initiation of the protocols. Scale bars, 300 μm (a) and 100 μm (b).

## 2. Peripheral devices to manipulate the chip

To meet the requirements of high-throughput applications, the liquid flow in the chip's biomimetic blood vessel was realized by a rocking shaker that generates a difference in liquid level height between the inlet and outlet, and the device is schematically shown in Figure S2a. The well distribution of the array chip is consistent with that of a commercially available 96-well plate, thus facilitating the sampling of the chip with a commercially available automated pipetting system （Figure S2b）.

**Citation:** Peng, X.; Wu, L.; Li, Q.; Ge, Y.; Xu, T.; Zhao, J. An Easy-to-Use Arrayed Brain-Heart Chip. *Biosensors* **2024**, *14*, x. https://doi.org/10.3390/xxxxx

Received: 16 September 2024
Revised: 10 October 2024
Accepted: 11 October 2024
Published: date

**Copyright:** © 2024 by the authors. Submitted for possible open access publication under the terms and conditions of the Creative Commons Attribution (CC BY) license (https://creativecommons.org/licenses/by/4.0/).

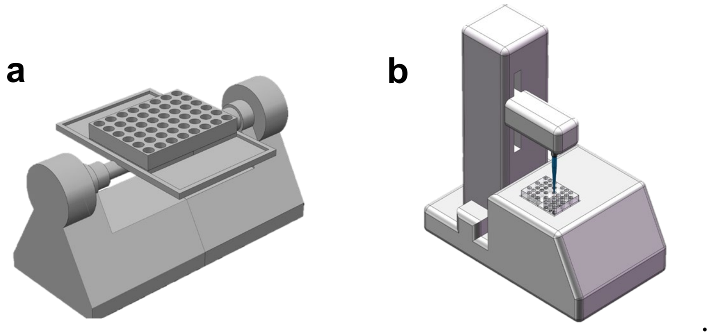


**Figure S2.** Schematic diagram of the brain-heart chip array peripherals. (a) A rocking shaker for perfusion. (b) An automated pipetting platform for chip sampling.

### 3. Surface Contact Angle Measurement

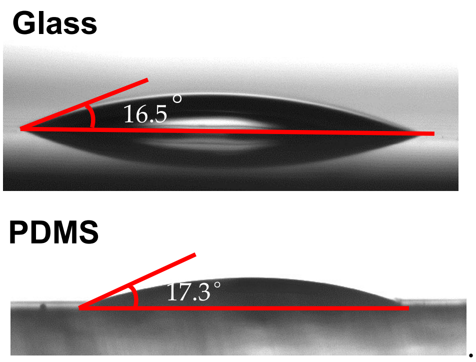


**Figure S3.** Contact angle measurement of a fibrinogen solution the the surfaces of plasma-treated glass and plasma-treated glass PDMS, respectively.

The static surface contact angles were measured using a contact angle analyzer (Dataphysics instrument, Germany) at room temperature and ambient humidity. The surfaces of PDMS or glass substrate were treated with plasma (200 W, 2 min) and then they were placed on ice for 1 minute, and then the surface contact angles were measured immediately. The fibrinogen solution at 10 mg/ml was placed in the ice before use. The contact angles of fibrinogen on plasma-treated glass and PDMS are 16.5° and 17.3° at 0°C, respectively, as shown in Figure S3.

### 4. Perfusion culture of HUVEC-RFPs alone in the chip

To test the stability of the endothelial barrier constructed on the fibrin, HUVEC-RFPs were primed into the vascular channel of the chip and incubated for two weeks. The fluorescence micrograph is shown in Figure S4. No cell invasion into the fibrin was observed.

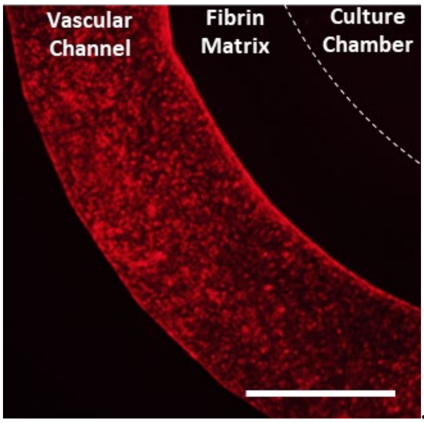


**Figure S4.** Fluorescence micrograph of HUVEC-RFP grown in the chip for two weeks. Scale bar, 1mm.

## 5. Video of fibrinogen solution spontaneously filled the hydrogel channel

The spontaneous filling of the hydrogel channel with a fibrinogen solution driven by capillary forces was recorded with a high-speed camera (CP70-1HS-C-1900-RT, Optronis GmbH, Germany). The video capture rate is 120 frames per second (fps), and the video playback rate is 1/16 of the true rate. Before fibrinogen entered, the hydrogel channel exhibits faint horizontal and vertical lines. This was attributed to maskless lithography, a photolithographic process that employs image splicing, resulting in SU8 mold with minute imprints at the splicing boundaries. Following the arrival of the fibrinogen at point P, a deceleration and subsequent re-acceleration process was observed. The discrepancy in the flow rate between the left and right branches of the fibrinogen flow was attributed to structural defects in the chip (bottom left of the video frame). However, this did not affect the structural integrity of the hydrogel barrier, which was constructed within 0.4 seconds.
